# Plastics and the microbiome: impacts and solutions

**DOI:** 10.1186/s40793-020-00371-w

**Published:** 2021-01-20

**Authors:** G. Lear, J. M. Kingsbury, S. Franchini, V. Gambarini, S. D. M. Maday, J. A. Wallbank, L. Weaver, O. Pantos

**Affiliations:** 1 School of Biological Sciences, University of Auckland, 3a Symonds Street, Auckland, 1010 New Zealand; 2 Institute of Environmental Science and Research, 27 Creyke Rd, Ilam, Christchurch, 8041 New Zealand

**Keywords:** Microplastics, Plastic pollution, Biodegradation, Plasticiser, Microbial community, Plastic additives, Bioremediation, Plastisphere, Toxic impact, Community dysbiosis, Rafting of pathogens and invasive species

## Abstract

Global plastic production has increased exponentially since manufacturing commenced in the 1950’s, including polymer types infused with diverse additives and fillers. While the negative impacts of plastics are widely reported, particularly on marine vertebrates, impacts on microbial life remain poorly understood. Plastics impact microbiomes directly, exerting toxic effects, providing supplemental carbon sources and acting as rafts for microbial colonisation and dispersal. Indirect consequences include increased environmental shading, altered compositions of host communities and disruption of host organism or community health, hormone balances and immune responses. The isolation and application of plastic-degrading microbes are of substantial interest yet little evidence supports the microbial biodegradation of most high molecular weight synthetic polymers. Over 400 microbial species have been presumptively identified as capable of plastic degradation, but evidence for the degradation of highly prevalent polymers including polypropylene, nylon, polystyrene and polyvinyl chloride must be treated with caution; most studies fail to differentiate losses caused by the leaching or degradation of polymer monomers, additives or fillers. Even where polymer degradation is demonstrated, such as for polyethylene terephthalate, the ability of microorganisms to degrade more highly crystalline forms of the polymer used in commercial plastics appears limited. Microbiomes frequently work in conjunction with abiotic factors such as heat and light to impact the structural integrity of polymers and accessibility to enzymatic attack. Consequently, there remains much scope for extremophile microbiomes to be explored as a source of plastic-degrading enzymes and microorganisms. We propose a best-practice workflow for isolating and reporting plastic-degrading taxa from diverse environmental microbiomes, which should include multiple lines of evidence supporting changes in polymer structure, mass loss, and detection of presumed degradation products, along with confirmation of microbial strains and enzymes (and their associated genes) responsible for high molecular weight plastic polymer degradation. Such approaches are necessary for enzymatic degraders of high molecular weight plastic polymers to be differentiated from organisms only capable of degrading the more labile carbon within predominantly amorphous plastics, plastic monomers, additives or fillers.

## Global plastic pollution

The first plastic to be produced in commercial quantities, Bakelite, was invented in the early 1900s. A scarcity of resources and a need to enhance technologies following the First World War drove the development of new and improved synthetic materials, including plastics. Plastics now constitute a large and diverse group of materials made from combinations of synthetic and semi-synthetic polymer materials, frequently incorporating additives which aid the manufacture and performance of the final product, such as plasticisers, antioxidants and flame retardants [1]. Plastics are predominantly derived from fossil fuels (e.g. oil or natural gas), although they may also be made from renewable resources (e.g. ‘bio-based’ plastics derived from corn starch or sugar beet); plastics such as polyethylene terephthalate (PET) may be synthesized from either source and are sometimes referred to as ‘drop-in’ plastics. With the onset of mass consumerism in the 1960s and a move away from the use of traditional natural materials to more versatile plastics, plastics are now an integral part of our everyday lives. Plastic production has increased exponentially since the 1950s, with an estimated 8300 million metric tonnes of virgin plastic being produced to date and an expected annual production rate of 1100 t by 2050 [2].

Despite the large variety of polymers available, just eight make up 95% of all primary plastics ever made, with polypropylene and polyethylene comprising 45% of global production [2]. The primary use of plastic is for packaging (36%), followed by use in building and construction (16%) [3]. Currently, the dominant polymer types are entirely fossil-fuel based and are not biodegradable in a timescale relevant for their end-of-life management. Fossil-fuel based biodegradable polymers such as polycaprolactone (PCL) and polybutylene adipate terephthalate (PBAT) are not currently used at large scale. In fact, less than 1% of polymers are bio-based, and of those 44.5% are ‘drop-in’ polymers which share the same properties of their fossil fuel-based versions, i.e., they are considered non-degradable [4]. Of the almost 360 million tonnes of plastic produced annually, only a small fraction (~ 1%) is bio-based [4].

At their end-of-life, there are essentially three fates for plastics: recycling; incineration and discarding. To date, end-of-life management of plastic products has not kept pace with rapid increases in production, resulting in widespread environmental contamination. Globally, it is estimated that only 10% of plastics are recycled and 14% incinerated; the remaining 76% goes to landfills or enters the natural environment [2]. Recent modelling estimates that under current rates of loss, with no changes to management practices and in conjunction with the anticipated increase in production, 710 million tonnes of plastic waste will have cumulatively entered the environment by 2040 [5]. Whilst large plastic waste normally comes to mind when discussing leakage to the environment, the natural wear and tear of items, such as ropes, clothing and tyres, sheds small fragments during use, facilitating the passive transport of smaller plastic fragments into the environment. These fragments, when less than 5 mm are referred to as microplastics, or nanoplastics if less than 1 μm [6]. Microplastic leakage is expected to increase by 1.3 – 2.5 times by 2040 under a business-as-usual scenario and equates to approximately 3 million trillion pieces [5]. This widespread ingress of plastics into the environment means they are distributed across the globe in many different forms and in all ecosystems so far investigated; from rivers and streams [7, 8] to deep ocean trenches [9, 10], mountain tops [11], and from the tropics [12] to the poles [13].

## Microbial impacts of global plastic pollution

The recent death of a Cuvier’s beaked whale in the Philippines with 40 kg of plastic waste in its stomach [14] and the necropsy of a young sperm whale on a Scottish beach yielding 100 kg of refuse [15] caught global media attention and scientists continue to report impacts of plastic waste on a wide range of species [16–18]. More than 800 animal species are already shown to have been affected by plastic pollution, and with an increasing number, from detritivorous sea snails [19] to apex marine predators [20, 21], being found to have internalised plastics. Globally, Wilcox, et al. [22] predict that as many as 90% of all seabirds ingest plastics. Post-mortem images of plastics spilling from the guts of dissected marine animals are causing us to reconsider unsustainable plastic use, yet the impacts of plastic pollution on most smaller organisms remain less well studied. Certainly, negative consequences of plastics have been reported for meiofauna such as *Daphnia magna* [23] and *Caenorhabditis elegans* nematodes [24], largely attributed to toxicological impacts, or blockage of the digestive system and related reductions in feeding rates. In contrast, the impact of plastics on environmental communities of microorganisms is rather less well researched.

**Fig. 1:**
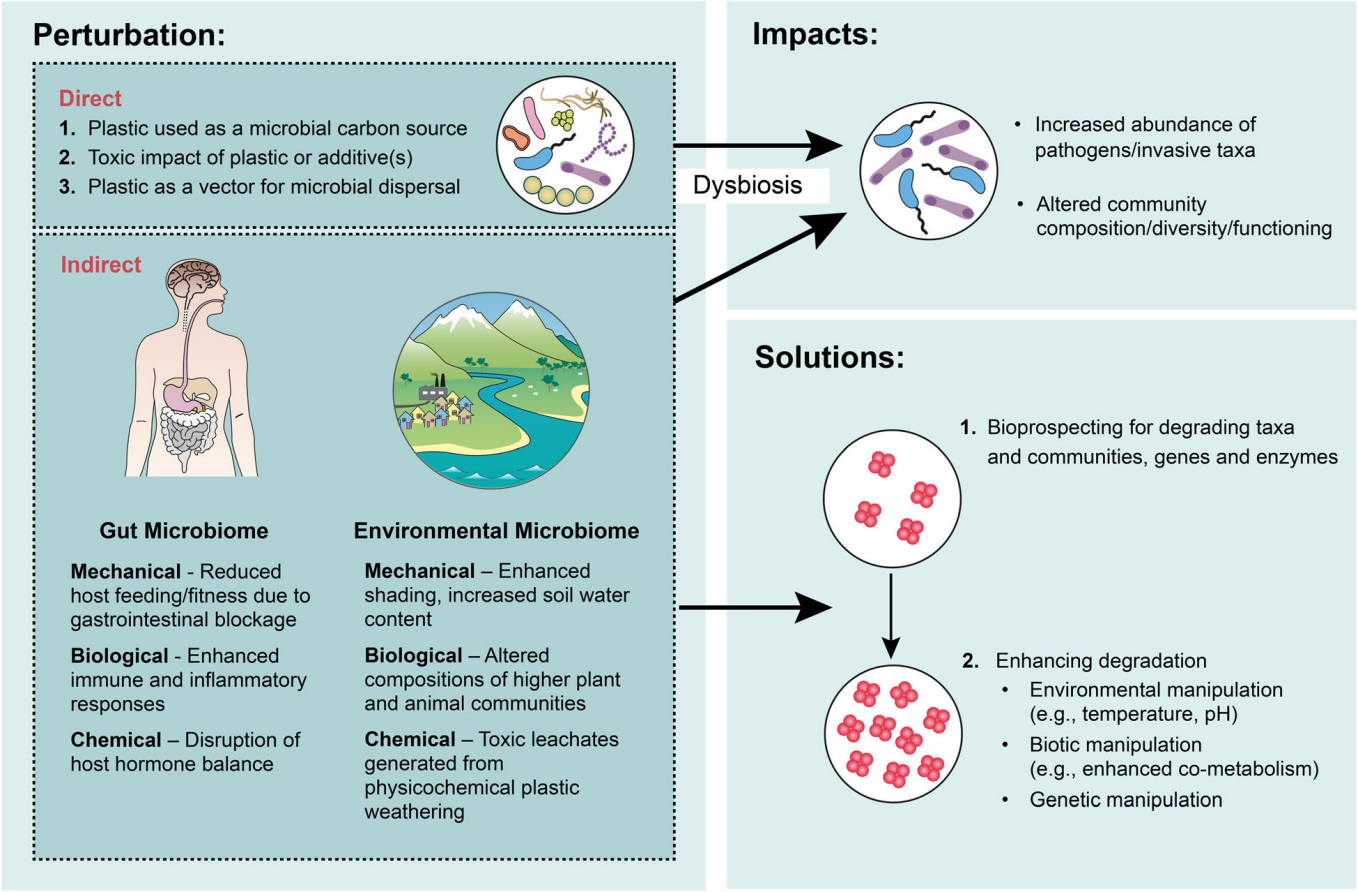
Schematic highlighting the diversity of direct and indirect impacts of plastics for gut and environmental microbiome communities and possible microbial solutions for the remediation of plastic waste

The term ‘microbiome’ describes the combined genetic material, or community, of microorganisms inhabiting a particular environment. While researchers continue to explore diverse microbiomes, including of soil, marine, freshwater, atmosphere and subsurface environments, the term ‘microbiome’ is perhaps predominently used to describe research into the microbiome of the gastrointestinal tract (the so-called ‘gut microbiome’). Since environmental plastics can concentrate in the digestive tracts of organisms from diverse trophic levels [25–27] they have the potential to impact the gut microbiome. However, due to their widespread environmental distribution, impacts of plastic pollution further extend to the microbiomes of diverse, non-host associated environments (which hereafter we refer to as the ‘environmental microbiome’). The direct impact of plastics on gut and environmental microbiomes are multiple (Fig. 1). (i) Some plastics and/or their associated additives *provide organic carbon* sources metabolizable by certain microorganisms. However, the microbial degradation of most plastics is restricted to only a few taxa [28], remains slow, and in many cases is unproven or disputed. Indeed, there remains a paucity of evidence for the microbial degradation of dominant plastic polymers, including polypropylene, polystyrene, polyethylene, nylon and polyvinyl chloride [29]. For these reasons, the impacts of plastics on microbial communities as a source of additional carbon are likely to be minimal, particularly in natural environments where alternative labile carbon and energy sources dominate. A notable exception to this may be following plastic consumption by certain insects where microbial degradation is postulated to be enhanced via ‘prior-processing’ by enzymes present within the gut [30]; this hypothesis however remains unproven. (ii) To a large degree, pure plastic polymers are chemically benign, having little *toxic impact*. However, industrial plastics contain additives including flame retardants (e.g., polychlorinated biphenyls and polychlorinated naphthalenes), plasticisers (e.g., bisphenol A) and UV stabilisers (e.g., benzotriazoles), some of which are demonstrated to impact microbial community composition and functioning. For example, plastic leachates from high-density polyethylene (HDPE) and polyvinylchloride (PVC) exert toxic effects on *Prochlorococcus* spp.*,* impairing cell growth and population density in a dose-dependent manner [31]. *Prochlorococcus* is among the most numerous of photosynthetic organisms on Earth [32], responsible for perhaps ~ 10% of ocean net primary production [33]; in this regard, plastic pollution has demonstrated potential to impact major global microbial processes. Consumption of plasticisers including bisphenol A [34] may similarly cause dysbiosis of the gut microbiome, impacting host health. (iii) Plastics may also change microbial communities by impacting rates and extents of dispersal, since they provide *a surface for microbial attachment* and thereby can aid the transport of microbial cells, including pathogens, both around the globe and into the gut. In comparison to these direct impacts of plastics on microbiomes, far less is understood about their indirect impacts. Plastics and their additives can impact the health of host organisms with consequences for the gut microbiota that is intrinsic to the wellbeing of higher animals [35].

In this review, we highlight recent knowledge on the direct and indirect impacts of plastics on the health and functioning of environmental microbiomes, including of the gut. We further consider how the impacts of plastics may be mitigated and also manipulated to enhance both rates and extents of plastic degradation.

## Impacts of plastics on the gut microbiome

Plastics cause a variety of undesirable mechanical, chemical and biological impacts on the species that ingest them. The consumption of plastics, either directly or via trophic level transfer [25], has multiple direct consequences, reducing appetite, impacting feeding activity and decreasing body weight [36], fitness [37] and fecundity [38]. In severe cases, the accumulation of large plastic masses may block the gastrointestinal tract; this has been recorded as a cause of death in diverse species including cetaceans [39, 40], turtles [41] and birds [42]. Smaller fractions of plastic may also bioaccumulate in the body, mostly in the gut, although translocation of plastics via the haemolymph and haemocytes of filter feeders is reported [26, 43], including to organs such as the liver and kidneys [44, 45]; this implies an ability for microplastics to cross the gut epithelial lining following ingestion and enter the circulatory system. Avio, et al. [43] explored the impact of polyethylene and polystyrene microplastics on the Mediterranean mussel (*Mytilus galloprovincialis*). Following 7 days of exposure to the plastic, histological analysis revealed aggregates of plastic in the intestinal lumen, epithelium and tubules. Further, increased DNA strand breakages provide evidence of genotoxic impacts, possibly caused by the greater production of reactive oxygen species (ROS) in response to microplastics. Nucleotide-binding oligomerization domain-like, or NOD-like receptor signalling pathways were enriched in *M. galloprovincialis* exposed to microplastics; these receptors recognise pathogenic factors entering the cell via phagocytosis and activate inflammatory responses. These findings support a growing body of evidence that micro- and nanoplastics cross biological barriers to promote immune and inflammatory responses [45, 46]. Where microplastics impact host immunity, this can further cause changes in gut microbial community composition and functioning. Oxidative states caused by inflammation can encourage the dominance of more resistant bacterial groups and, if associated with a rise in anaerobic respiratory terminal electron acceptors, may support the growth of anaerobic taxa such as members of the Enterobacteriaceae [47]. The gut microbiome influences not only the host immune system, but also contributes to digestion and the provision of essential nutrients [48], the degradation of harmful substances [49] and pathogen control within the gut [50]. The consumption and translocation of microplastics among bodily tissues therefore has far reaching consequences for the homeostasis normally maintained between a host and its microbiome.

While the physical presence of plastics demonstrably impacts the microbiome-gut-immune axis, additives which leach from plastic polymers have further consequences. Plasticisers are the largest group of plastic additives [51], particularly phthalates which may concentrate in bodily tissues to induce multiple adverse effects. For example, diethyl-hexyl phthalate (DEHP) causes antiestrogenic properties in fish hindering the development of reproductive organs [52], presumably due to competition with endogenous oestrogens for the receptor, and dibutyl phthalates delay gonad development and functioning in mammals [53] and amphibians [54]. The presence of bisphenol A (BPA) in the environment is predominantly due to it being a constitutive monomer of polycarbonate plastics, although it is also commonly added to PVC as a plasticiser. BPA has feminising impacts in fish, reducing male sperm quality, delaying and inhibiting ovulation in females [55] and in cases of high-concentration exposure, can induce intersex states [56]. Impacts on many other organisms are reported; BPA influences thyroid functioning and larval development in amphibians [57], early embryo development in marine bivalves [58] and reproductive birthweights and altered oestrous cyclicity in mammals [59, 60]. Plastics also adsorb organic pollutants such as polychlorinated biphenyl (PCB) from their environment [61, 62]; these contaminants may be transferred to the biological tissues of organisms such as birds following plastic ingestion [51]. While concentrations of plastic-associated contaminants are unlikely to be a major contributor to environmental concentrations of contaminants such as PCBs [63], a variety of plastic-associated compounds must be considered when assessing the impacts of plastic pollution on host-microbiome interactions [64].

The impacts of plastic additives on the gut microbiome remains little explored, although Adamovsky, et al. [65] assessed the consequences of environmentally relevant concentrations of the widely used plasticiser DEHP [66] on zebrafish. DEHP caused dysbiosis of the gut microbiota [67], and assessment of the gastrointestinal transcriptome revealed the up-regulation of T cells thought to play key roles in pathogen neutralisation by maintaining the integrity of the intestinal epithelia, while downregulating neuropeptide Y, a hormone which can modify immune activity by regulating T cell function. Analysis of the gut microbiome implicated several microbial metabolites that may contribute to immune and intercellular communication, including decreased L-glutamine in males and D-fructose 6-phosphate in females. Following DEHP exposure, Adamovsky, et al. [65] thereby identified the impact of microbial bioactive metabolites on host immune system dysregulation. Further negative impacts are reported. For example, the abundance of *Mogibacteriaceae*, *Sutterella* spp. and *Clostridiales* bacteria is increased within female mice exposed to BPA [68], presumably due to disrupted regulation of the sex hormones testosterone and oestrogen, implicating BPA for causing sex-dependent changes in the gut microbiome. The exposure of animals to plasticisers and plastic precursors including BPA are confirmed to impact intestinal microbial profiles in multiple studies [69–71], sometimes favouring microbial markers of dysbiosis such as a community dominance by Proteobacteria [72]. Nevertheless, understanding of cause and effect in host-microbiome interactions remains limited.

As we will later describe, microplastics are potential vectors of pests and pathogens around the globe via ocean currents, but so too may they vector pathogens into the gut. Microbial attachment to plastic particles can enhance both microbial dispersal and survival, as biofilms offer protection from environmental stress and enhanced opportunities for the sharing of beneficial traits via horizontal gene transfer. Pathogens such as *Vibrio parahaemolyticus,* which causes septicaemia and gastroenteritis in humans, have been identified in marine plastic-associated biofilm communities [73] and ingestion of such organisms hitchhiking on plastics might cause disease. However, even if not pathogenic, ingested organisms can influence gut community composition if they are capable of competing for resources within the gut [74]. Although the rich taxonomic and functional diversity of ‘plastisphere’ microbial communities has recently been unveiled [75], the role of plastics for microbial dispersal and colonisation of the gut remains poorly studied and understood.

## Impacts of plastics on the environmental microbiome

In terrestrial environments, the mere presence of plastics exerts physical impacts directly impacting microbial communities. For example, agricultural plastic mulch films applied to enhance short-term crop productivity cover perhaps ~ 20 million hectares of farmland worldwide [76] and are a significant source of terrestrial plastic contamination [77]. While most research has focused on the impact of synthetic plastic films, the microbial consumption of biodegradable plastics is noted to have profound impacts on soil microbial communities [78]. Once embedded in the soil, plastics impact soil-water interactions by increasing water content [79], a major determinant of soil microbial community composition and functioning [80, 81]. By altering the availability of water, the physical impact of plastics on the soil environmental microbiome may be substantial [82]; the consequence of other physical impacts, such as increased shading by plastics which has been hypothesised to reduce aquatic photosynthesis, remain largely unsupported [83, 84].

The presence of plastic has direct chemical consequences for environmental microbial communities. Readily biodegradable plastics such as polylactic acid (PLA) contribute available carbon and in some cases significantly increase microbial biomass and enzyme activity [85]. The presence of such plastics in soils alter community composition, enriching the abundance and activity of certain taxa (e.g., members of the Ascomycota fungi [86]). The impact of more recalcitrant plastics remains less well understood, although even where degradation is slow, plasticising agents and additives such as phthalate acid esters may nevertheless leach, reaching elevated concentrations within receiving environments [87] and cause significant shifts in microbial community composition, abundance and enzyme activity [88, 89]. Although plastic additives are not always observed to impact environmental microbiomes at environmentally relevant concentrations [90], the sheer diversity of plastic additives used [91] means their impacts are yet to be fully understood. Of particular interest, Tetu, et al. [31] investigated the consequences of plastic leachate from HDPE bags and PVC matting on marine *Prochlorococcus* and confirmed that exposure to even the lowest dilution (approximately 1.6 g L^− 1^ and 0.125 g L^− 1^, respectively) of HDPE and PVC from 5-day old leachate impaired *Prochlorococcus* growth*.* Further, the transcription of genes associated with primary production was highly impacted, indicating that exposure to leachate from common plastic items has the capacity to impair the photosynthesis of the most dominant marine organisms.

Through the ubiquitous interactions between microorganisms and macroscopic plants and animals [92, 93], plastics and their associated compounds exert multiple indirect biological impacts on environmental microbiomes. For example, plants can be impacted as they take up plastics such as polystyrene via their roots, altering root length, weight and oxidative stress responses, possibly by the disruption of cell wall pores and cell-to-cell connections used for nutrient transport [94, 95]. Plant taxonomy and health play an important role in shaping soil and rhizosphere microbiomes, impacting the quantity and quality of root exudates [96] and the potential of plants to recruit specific members of the soil microbiome and promote the expression of genes, including those required for chemotaxis and biofilm formation [97]. Where observed, the impacts of plastics on the composition and health of plant and animal communities will likely have significant influences on environmental microbiomes, but to date insufficient evidence exists to suggest a strong link. Impacts on macroorganisms are rarely detected at environmentally relevant concentrations of microplastic; Judy, et al. [98] found no evidence of any impact of microplastics on wheat seedling emergence and production, or on the mortality or behaviour of earthworm and nematode populations.

While much research has focused on the impacts of plastics on microbial communities in situ*,* environmental plastics also influence rates and extents of microbial dispersal among environments. Buoyant plastics such as polyethylene, polypropylene and polystyrene, are transported over long distances by winds and oceanic currents [99] whereas non-buoyant plastics such as PET and PLA may act as a vector to transport surface-associated microbes to deeper water [100]. Microbial groups, including toxic microalgae [101] and potential human [75] and animal pathogens [102] have been detected associated with marine and freshwater plastics [73, 103] along with diverse antibiotic-resistant taxa [104]. Plastics are further postulated to vector pathogens through wastewater treatment plants [105] and pest species via ballast water [106]. Microbial communities colonising environmental plastics likely aid larval settlement and colonisation by species including bryozoans and polychaete worms, thereby assisting the movement of invasive marine macroorganisms around the globe [107]. Thus, in addition to supporting or retarding the growth of certain taxa, environmental plastics likely play significant roles in the dispersal of both microbes and higher organisms across diverse spatial scales and habitat types. Interestingly, the microbial colonisation of plastics can also impact particle buoyancy and transport [108, 109].

## Assessing diverse plastisphere communities via amplicon and metagenome DNA sequencing

The development of molecular methods, including high-throughput DNA sequencing technology, is increasing our knowledge of the diverse nature of plastic-associated microbiomes. Although no taxa are known to only, or even to predominantly colonise plastic surfaces, multiple studies have demonstrated how the microbiomes of plastic debris differ from those present in the surrounding environment [110–113], with an overrepresentation in the plastisphere of bacterial phyla such as the *Proteobacteria, Bacteriodetes* [114] and *Cyanobacteria* [115] and fungi such as *Chytridiomycota* [113]. Nevertheless, with studies on the community composition of plastisphere microbiomes still in their infancy, it remains unclear the extent to which a core plastisphere community exists and the degree to which this differs from comparable microbiome communities in the same environment.

The specificity of plastisphere communities has been investigated in comparison to communities growing on inert surfaces such as glass and ceramic with varying results. A study by Oberbeckmann, et al. [116] using 16S rRNA gene amplicon sequencing for taxonomic analysis found no significant difference between the pelagic microbial communities associated with PET plastic bottles and glass microscope slides (as a control) deployed for 5-6 weeks. Pinto, et al. [117] also found that the overall community assembly on glass was similar among biofilms developing on HDPE, LDPE and PP over a period of up to 2 months, with families such as *Flavobacteriaceae, Phyllobacteriaceae, Planctomycetaceae* and *Rhodobacteraceae* being highly abundant across all surfaces. Such findings (also see Dang, et al. [118]) lead us to assume that there may be no specific plastic-associated communities. However, despite finding no differences in the total composition of communities growing on glass, HDPE, LDPE and PP (noting that significant differences were however observed for communities on PVC), Pinto, et al. [117] identified a subset of these communities incubated after immersion into seawater for up to 2 months, which was nonetheless responsive to the characteristics of individual plastic polymers or their additives (also see Ogonowski, et al. [119] and Kelly, et al. [7]). A higher relative abundance of the bacterial family *Rhodobacteraceae* discriminated communities growing on HDPE and *Sphingomonadaceae* for communities growing on LDPE, as compared to glass. Using a longer period of incubation, Kirstein et al. [120] found that after 15 months in a natural seawater flow-through system, biofilms from HDPE, LDPE, PP, PS, PET, PLA, styrene-acrylonitryle (SAN), polyurethane prepolymer (PESTUR) and PVC were significantly different to communities formed on glass. While communities on PVC were noticeable for having a high abundance (> 5%) of the bacterial genus *Flexithrix,* differences in the abundances of other plastic-specific taxa were largely attributed to variation in the presence and abundance of less dominant OTUs, suggesting that rarer species form specific associations with certain plastic types [121]. Also supporting the notion that less dominant members of the community may respond more specifically to the presence of different plastics, Erni-Cassola, et al. [122] demonstrated that during two-day incubations, weathered LDPE was enriched with a distinct community (particularly members of *Roseobacter-, Oleiphilus-* and *Aestuariibacter-*like taxa) from untreated PE and glass. However, this distinction was not detectable after 9 days, suggesting that substrate-specific microbes present in the plastisphere are quickly masked as the community matured and putative plastic-specific taxa were outnumbered. Interestingly, while significant differences in microbial community composition are not consistently reported among communities developing on different plastics, different plastic colours have recently been implicated as a significant determinant of plastisphere microbial community structure and functional diversity [123].

To date, a majority of studies assessing the formation and development of plastisphere communities have been conducted in the laboratory using different types of plastic of various condition (e.g., from ‘virgin’ plastics specifically manufactured for a study [124] to post-consumer plastics such as discarded bags and PET bottles [116]). Considering the longevity of plastic debris in the environment, the relatively short lengths of most lab-based studies may not be enough to explore the full degradative potential of the plastisphere microbiome. Environmental plastics hosting mature plastisphere microbiomes provide an alternative way to investigate the many factors that can influence plastisphere formation, such as plastic composition, age and condition. However, characterisation of aged microplastics, which dominate the marine plastisphere in terms of abundance, is often restricted as the biomass recovered from environmental microplastics is frequently very low, limiting abilities to recover sufficient nucleic acids for sequence analysis. As a consequence, there remain many unanswered questions regarding the plastisphere of aged environmental microplastics in particular.

As our knowledge of microorganisms present in the plastisphere is growing, there are still important questions that remain unanswered. (i) Which microorganisms act as pioneer species when the plastic is first introduced into the environment, and do the priority effects of early colonisation affect the overall composition and metabolic potential of the microbial community later on? These questions are of particular importance since the enrichment of plastic-degrading organisms may predominantly occur during early stages of colonisation, before the labile substrates generated from weathering are depleted and these plastic-specific microbes are dominated by more generalist biofilm-dwelling taxa [122]. (ii) Does there exist a core global community of plastic-degrading taxa, or do they exhibit substantial geographic or habitat-specific biogeography? (iii) If core members of the plastisphere vary in abundance between plastic types and biofilm maturity, can the presence and abundance of certain microorganisms indicate the approximate type and age of plastic debris? Answers to these questions will assist our ability to identify plastic-specific microorganisms from different regions, biomes, on different plastics and at different stages of plastic aging and degradation. Additionally, such knowledge likely increases our ability to use microbial community DNA to inform on the environmental impact of plastics (for example by adopting the approach of Hermans, et al. [125]).

As highlighted by Wright, et al. [126], many studies have characterised the plastisphere through taxonomic analyses [112, 117, 121, 122], however, there remains a lack of knowledge surrounding the functional potential of these communities. Bryant et al. [115] were among the first to explore the metabolic potential of the plastisphere microbiome using shotgun metagenomics, hypothesising that the genomes of plastic-associated taxa would be more distinct and exhibit increased metabolic activity compared to free-living bacteria in the surrounding marine water. Compared to those of the picoplankton community, their study revealed an increased abundance of genes encoding for chemotaxis and nitrogen fixation as well as several putative genes for xenobiotic biodegradation in plastic-associated communities. This included a gene encoding for 2,4-dichlorophenol 6-monooxygenase, a hydroxylase associated with the degradation of chlorinated aromatic pollutants [127] sometimes produced from polymer and plastic additive pyrolysis [128]. Similarly, the study revealed an increased abundance of multiple genes encoding for ring-cleaving enzymes, such as protocatechuate 3,4-dioxygenase and particularly homogentisate 1,2-dioxygenase, previously linked with styrene and polycyclic aromatic hydrocarbon degradation [129]. Whilst Bryant, et al. [115] were unable to confirm if microbes within the plastisphere are able to degrade the plastic polymer, the increased abundance of genes encoding for the degradation of several xenobiotics may assist identification of new plastic-degrading enzymes, and also the taxa expressing and utilising these enzymes. In common with previous studies, Pinnell & Turner [130] found the community composition of fossil fuel-derived PET-associated biofilms to be indistinguishable from those growing on ceramic beads deployed at the sediment-water interface of a coastal lagoon; in contrast, microbial communities associated with bio-based PHA pellets were dominated by sulphate-reducing organisms. Metagenomic analysis of the bioplastic-associated communities revealed substantial phylogenetic diversification of one depolymerase in particular, polyhydroxybutyrate (PHB) depolymerase, alongside an almost 20-fold increase in abundance of the depolymerase genes, suggesting they are widely distributed within the biofilm. An increased abundance of genes associated with sulphate reduction and plastic degradation, such as depolymerases, esterases and sulphate reductases, were also reported. Thus, while bio-based plastics continue to be perceived as an environmentally friendly alternative, if sedimentary inputs are large enough, the authors speculate that microbial responses could impact benthic biogeochemical cycling through the stimulation of sulphate reducers.

It is likely that communities work together to access plastic-derived carbon; the genes encoding for the degradation of alkanes, for example, are distributed among diverse assemblages of hydrocarbonoclastic organisms [131]. A greater understanding of the dynamics of plastic-associating communities may be achieved by determining co-occurrence patterns and associations among different organisms and genes. Toxic and poorly labile carbon substrates have been observed to strongly favour facilitation among microbial species such that they can each grow and degrade these substrates better in order to survive [132]. Where taxa or gene products are presumed to play a beneficial role in plastic degradation, correlated increases in their abundance across multiple samples as indicated by network analysis (e.g. see Gatica, et al. [133]) might identify other organisms and molecular pathways that could benefit from the community response to plastic contaminants.

## Mitigation of plastic pollution by the gut microbiome

**Fig. 2:**
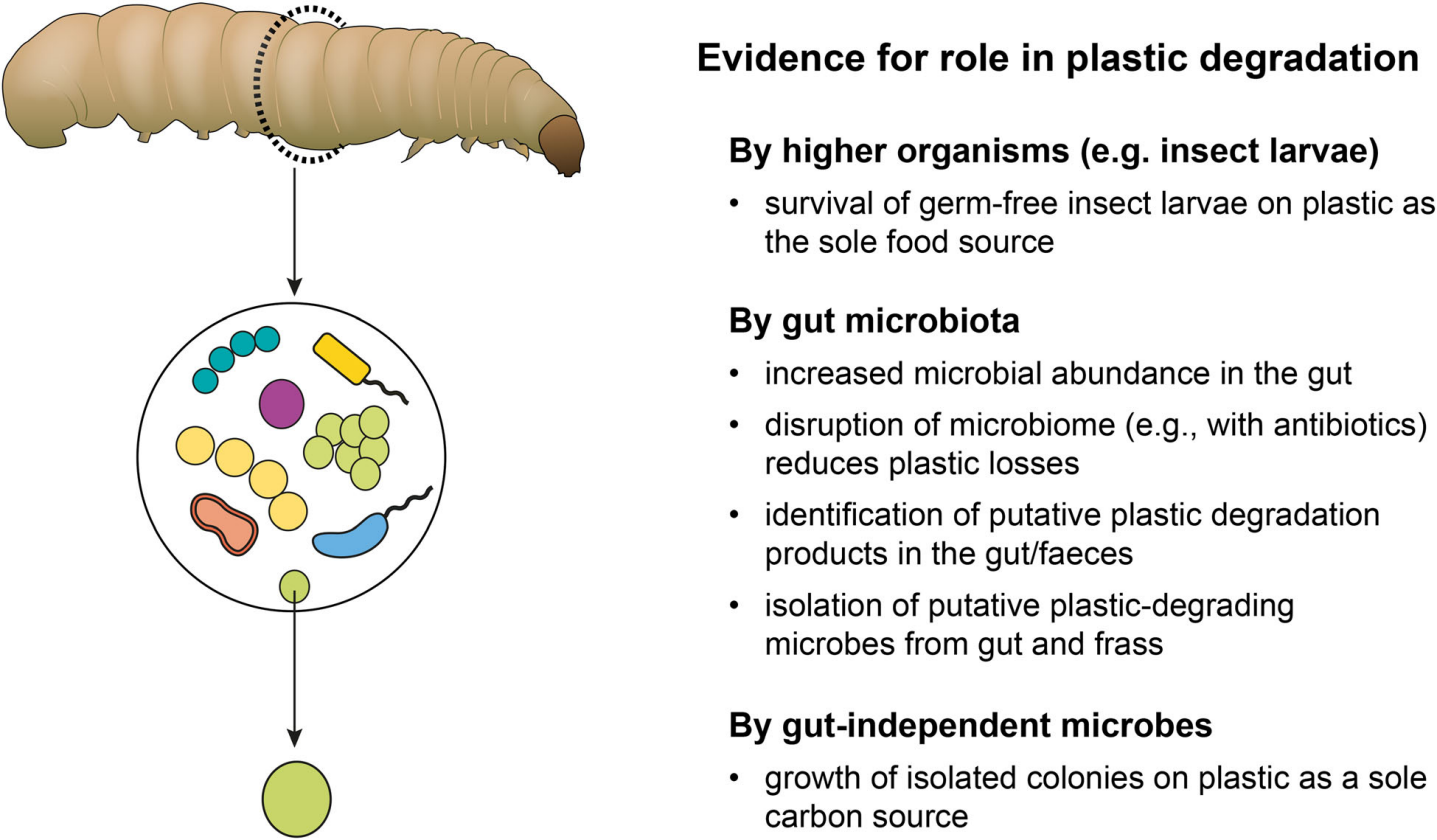
Evidence for a role for insects, host-associated microbes, or host-independent, free-living microbes in plastic degradation. Degradation of the plastic polymer may be detected by a variety of methods, including: [i] mass loss of plastic such as clear zone development around colonies on plastic-infused/overlaid agar, [ii] altered plastic surface properties (e.g., visible by scanning electron microscopy) and [iii] generation of degradation products (e.g., CO_2_, polymer metabolites detected by Fourier-transform infrared spectroscopy or high-performance liquid chromatography)

Recently, several insect species (particularly the larvae of darkling beetles, wax moths and meal moths) have garnered interest for their ability to consume and degrade a diversity of plastic polymers. For example, larvae of the Indian meal moth *Plodia interpunctella* can ingest and appear capable of degrading polystyrene [134] as do larvae of yellow and giant mealworms *Tenebrio molitor* and *Zophrobas morio*, respectively [135, 136]. Larvae of the greater [137] and lesser wax moths (*Galleria mellonella* and *Achroia grisella* [138]) are similarly reported to degrade polyethylene and polystyrene, respectively. Isotope analysis provides evidence that carbon from plastics such as PE is incorporated into the biomass of invertebrates [139]. Despite the findings of these and other studies, it nevertheless remains uncertain the extent to which either the higher organism or its associated microbiome contribute toward plastic polymer degradation. Further, the extent to which these biodegradative processes may be accelerated by synergistic effects of the host-microbiome remains unclear (Fig. 2).

Many organisms consume plastic incidentally and gain no nutritional value from its consumption; plastic has been found in abundance within the guts of diverse organisms from seabirds [22] and fish [140] to marine and freshwater worms [36, 141] and zooplankton [142]. Although the ingestion of plastics by species including the common earthworm *Lumbricus terrestris* is associated with reductions in plastic size distribution [143], in many cases, demonstration of plastic degradation, e.g. by conversion to CO_2_ or incorporation of plastic-associated carbon into animal biomass, is unsubstantiated [144]. Similarly, the ‘consumption’ of plastics by mealworms and wax moth larvae has gained much attention [30, 145], but confirmation of plastic degradation by the hosts’ gut-derived enzymes, independent of the hosts’ microbiome, requires further confirmation [146]. In most cases, it remains to be seen whether the host derives any nutritional benefits from plastic as a source of energy; without stronger evidence of more complete degradation in the gut, plastic fragments may merely be generated via mechanical processes (e.g. chewing) and ejected into the environment. To confirm plastic degradation by macroinvertebrates, studies in germ-free organisms (i.e., those lacking a microbiome) are desirable, noting the physiological homeostasis of organisms such as *T. molitor* are impacted by related changes in digestive enzyme expression by axenic cultures [147]. Another approach is to track the fate of radiolabelled (e.g. ^13^C, ^14^C) plastic polymer via incorporation into the cellular biomass or respiration products of consumer invertebrates [139], preferably in the absence of host microbial taxa to also eliminate the possibility of trophic carbon transfer. The lack of evidence to date for plastic degradation by germ-free larvae instead supports that microbiota are important drivers of plastic degradation within the invertebrate gut.

Since diverse putative plastic-degrading microbial taxa have now been described, including isolates from gut microbiota [28], it is hypothesised that the enzymes of gut-associated microbial taxa, rather than the enzymes of the host per se, perform most, if not all, plastic degradation by plastic-consuming invertebrate taxa. In a series of experiments, Cassone, et al. [148] provide multiple lines of evidence for the degradation of LDPE by the intact microbiome of *G. mellonella* larvae. The larvae of *G. mellonella* readily consume beeswax, which in some aspects is similar to plastics such as PE, being comprised of a diverse mixture of long-chain hydrocarbons. Hence, plastic consumption propensity may be related to the structural or chemical similarity of plastics to their preferred food source. PE-fed caterpillars had a far greater abundance of gut-associated microorganisms as compared to starved individuals, or even to organisms fed a natural diet of honeycomb, suggesting their microbiota could benefit from the abundance of PE in the gut. Antibiotic-treated caterpillars fed PE also excreted only half the concentration of ethylene glycol compared to untreated animals. Since ethylene glycol is a putative by-product of PE metabolism [30] this was used to imply a direct role of the gut microbiome for PE degradation. The inhibition of plastic depolymerisation following antibiotic treatment has now been observed in numerous studies, indicating that the host organism alone is poorly able to utilise plastic as a carbon or energy source, or is at least in part reliant on its microbiome as a source of plastic-degrading enzymes [135, 136, 144, 148]. Providing further evidence for a microbial role in plastic degradation, Cassone, et al. [148] isolated and grew bacteria from the gut (identified as *Acinetobacter* sp.) on carbon-free media, supplemented with PE fragments. A further observation was that the *Acinetobacter sp.* was only capable of degrading plastics at a very slow rate when isolated from the gut, providing evidence that plastic degradation is maximised by synergisms occurring between the host and its gut microbiome community, although the importance of community microbial interactions cannot be disregarded. Nevertheless, the extent to which the larvae impact the structure of the plastic polymer or associated additives, or enhances beneficial functional attributes of its gut microbiota currently remains unclear.

Prior to the study of Cassone, et al. [148], multiple authors had already isolated putative plastic-degrading bacteria from the insect gut microbiome. Yang, et al. [144] isolated the bacterium *Exiguobacterium* sp. Strain YT2 from the gut of styrofoam-fed mealworms and demonstrated its ability to grow on polystyrene film as a sole carbon source, associated with changes in the surface topography and hydrophobicity of the plastic. Mass loss of polystyrene combined with decreases in molecular weight and the release of water-soluble degradation products were used as further evidence to highlight the capacity for gut-associated microbes to degrade plastics (noting that Danso, et al. [29] question if sufficient evidence is available to confirm degradation of the high-molecular weight polymer, i.e. the polystyrene itself, rather than styrene monomers incorporated within the polymer matrix). Similar studies implicate *Aspergillus flavus, Bacillus* sp. YP1 and *Enterobacter asburiae* YT1 isolated from insect gut microbiomes as being capable of PE degradation [134, 149]. While such findings identify a possible role for gut-associated microbes to degrade plastic, organisms isolated from non-host environments are similarly capable of plastic degradation and could be exploited for their biodegradation capacity.

## Mitigation of plastic pollution by the environmental microbiome

**Fig. 3:**
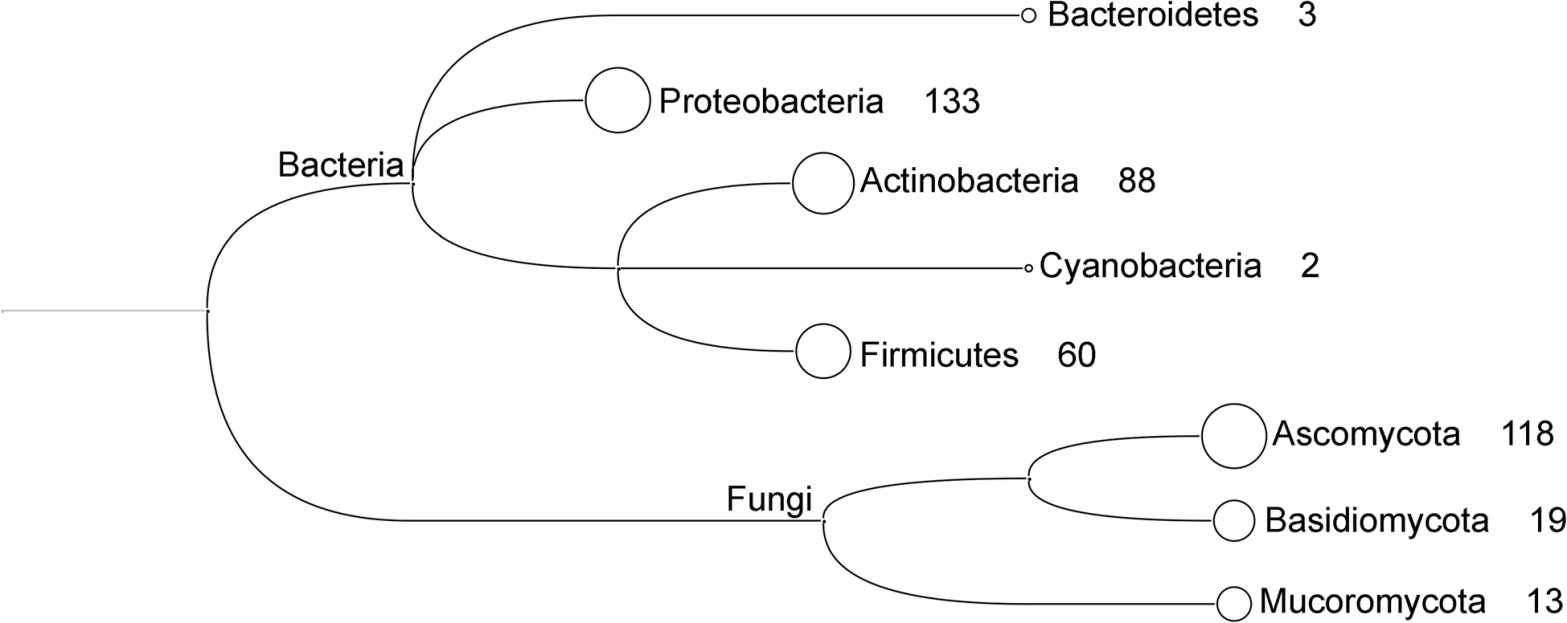
Number of putative plastic-degrading organisms reported by Gambarini, et al. [28], classified at the level of phylum level. The number following the phylum name represents the number of species from that specific phylum that are reported as plastic-degraders

The first evidence that free-living environmental taxa contribute to plastic degradation was only published circa 30 years after the first commercial plastic production, in 1974, when Fields, et al. [150] showed that the fungus *Aureobasidium pullulans* was capable of PCL degradation. Since then, the number of microorganisms suggested as capable of plastic biodegradation has increased considerably. A recent study by Gambarini, et al. [28] reports over 400 publications describing the degradation of 72 different plastic types by 436 species of fungi and bacteria. Presumptive plastic-degrading microbes identified to date belong to five bacterial and three fungal phyla. Among the bacterial phyla, Proteobacteria (*n* = 133), Actinobacteria (*n* = 88), and Firmicutes (*n* = 60) have the greatest number of reported species, while Bacteroidetes (*n* = 3) and Cyanobacteria (*n* = 2) have far fewer. The fungal phyla include Ascomycota (*n* = 118), Basidiomycota (*n* = 19), and Mucoromycota (*n* = 13) (Fig. 3).

As outlined earlier, a small number of plastic-degrading microbes have been isolated from plant- and animal-associated microbiomes [149, 151, 152]. However, most isolates reported in the literature were derived from soil [153, 154] or from waste processing sites such as composting facilities [155] and landfills [156]. An additional source comprises bacteria and fungi already deposited in culture collections [157]. All major synthetic polymers have species reported to degrade them, for instance PE [158, 159], PET [160, 161], PP [162], PS [163], PU [164] and PVC [165]. However, the strength of evidence for degradation varies by plastic type. To date, PET biodegradation has been studied the most comprehensively. A notable example includes the PET-degrading bacterium, *Ideonella sakaiensis*, isolated from sediment in the vicinity of a Japanese bottle recycling plant [161]. *I. sakaiensis* is the first organism for which the degradation of PET was well-described and the enzymatic degradation of PET elucidated, characterised [166] and enhanced [167]. Conversely, there is only weak evidence for the biodegradation of synthetic polymers such as nylon, PP, PS and PVC. For instance, nylon-oligomer biodegradation by the bacterium *Agromyces* sp. KY5R has been shown by Yasuhira, et al. [168] and the genes and corresponding enzymes responsible for the biodegradation activity have been identified; however, biodegradation of the plastic polymer (i.e. not just monomers and oligomers) is yet to be confirmed.

## Bioprospecting for novel mechanisms of plastic degradation

**Fig. 4:**
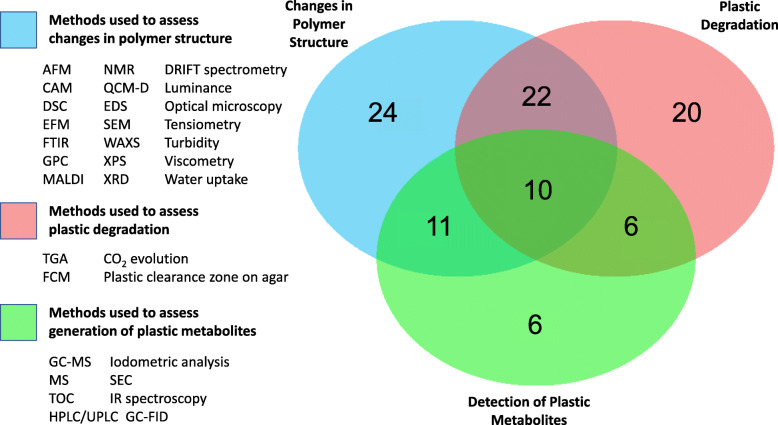
Percentage of studies using evidence for plastic degradation by microbial species based on: (i) changes in polymer structure (blue), (ii) physical loss of plastic mass (red), or (iii) detection of plastic metabolites (green), or these techniques in combination. Data were compiled using the

Currently, there is a lack of information necessary to critically validate many reports of plastic degradation by microbial taxa or communities or to accurately reproduce the research. For instance, many reports provide no information regarding polymer composition and omit details of fillers and additives that may be present in polymer composites. Therefore, it is frequently not possible to differentiate between the microbial degradation of plastic polymers or their additives. The strength of the degradation evidence is also greatly dependent on the techniques applied, which can be divided into three main categories, those detecting: (i) changes in the polymer structure, (ii) physical loss of plastic mass and (iii) the generation of plastic metabolites. The strongest evidence of plastic biodegradation is likely achieved using a combination of techniques from all three categories. However, analysis of the dataset of Gambarini, et al. [28], which compiled data from 408 studies, revealed that of the microorganisms reported to degrade plastics, 48% of reports were based on assays relating to only one of these categories, 39% used techniques that covered two categories, and just 10% used techniques that covered all three (Fig. 4).

Most reports of plastic degradation by microbial isolates do not go on to explore the genes and enzymes responsible for the reported activity. In fact, only around 14% of the microorganisms reported to degrade plastic have the gene sequences conferring the degradation activity elucidated [28]. This represents a major shortcoming since knowledge of the relevant biochemical and molecular data provides the capability to advance the plastic biodegradation field enormously, allowing the search for new putative plastic-degrading genes in novel microbiomes by comparison to enzyme data banked in structural and molecular databases. Crucial information and procedures related to the reported plastic degraders are frequently missing or incomplete in the current literature, for example, the location and conditions of isolation of the plastic-degrading isolate, strength of evidence for degradation, accurate taxonomic classification, and a lack of deposited strains in culture banks. By not addressing these points adequately, reports of plastic degradation, possibly in a majority of studies undertaken to date, must be treated with caution.

To exploit the broad phenotypic diversity that may already be present in natural populations, future advances in plastic biodegradation will likely benefit from isolation of novel microorganisms from diverse microbiome communities. This calls for consideration of the sampling environment and likely growth requirements of organisms within the microbiome, the plastic type of interest and the empirical tests required to delineate growth-linked biodegradation of the polymer. By reviewing the current literature, we provide a ‘best practice’ workflow of methods necessary to describe the pathways of growth-linked plastic biodegradation, beginning with appropriately characterising the plastisphere microbiome and concluding with the identification of plastic biodegradation genes and pathways (Table 1).

**Table 1:** Best practices for reporting microbial plastic degradation. We describe information, techniques, and practices that are critical to provide strong evidence for biodegradation, as well as steps necessary to maximise reproducibility of the findings

Item	Description	Importance	Best practice examples
Plastic identity	Descriptive name for the polymer, molecular weight and source.	Study reproducibility.	Almeida, et al. [151, 160]
Plastic composition	Complete polymer composition, plus composition and quantity of all additives and fillers.	To differentiate the degradation of polymer and additives.	Montazer, et al. [169], Novotný, et al. [170]
Microbial taxonomic classification	Taxonomic classification from well-characterised marker genes such as full 16S rRNA gene sequences for bacteria.	Reproducibility and the extrapolation of findings to related species. Benefits studies into the phylogenetic distribution of plastic-degrading traits.	Novotný, et al. [170], Hu, et al. [171]
Isolation environment and conditions	Strain isolation location and site-specific properties such as temperature and pH.	Identification of environments favourable for microbial plastic degradation.	Novotný, et al. [170]
Strain accessibility	Deposition and description of isolated strains in international culture banks.	Permits greater reproducibility and further study by other researchers.	Yoshida, et al. [161]
Assessment of plastic degradation	Description of techniques used for confirmation of degradation, and preferably the use of multiple complementary methods (Fig. 4).	Confirmation of degradation. It is important to confirm how techniques differentiate between the degradation of the polymer and additives, where included.	Yoshida, et al. [161, 164]
Plastic-degrading enzyme and gene identification	Identification of the enzyme responsible for the biological degradation and its gene sequence.	Allows mining of molecular databases, recombinant gene expression, enzyme optimisation, etc.	Kawai, et al. [160], Yoshida, et al. [161]

Based on protein mutagenic and structural analysis studies [166], alongside homology database searches [28], it is likely that certain microorganisms already possess plastic degradation genes but do not express them in situ, and/or derive energy from more readily utilisable carbon sources when available. By incorporating inert controls (e.g., glass or ceramic surfaces), we may be able to distinguish between genes acquired and expressed for the process of plastic-degradation, from those normally expressed in biofilm communities (i.e. including where plastic is not present). Yoshida et al., [161] demonstrated that *I. sakaiensis* possesses two genes encoding enzymes which degrade PET (*Is*PETase and *Is*MHETase). However, they did not address if the *Is*PETase might be used by the organism for other functions, or whether it was being used in situ to degrade PET within the PET recycling plant from which the organism was originally isolated. Structural analyses of the *Is*PETase revealed that the enzyme has a wider active-site cleft compared to ancestral cutinase homologs [166]. Narrowing the active-site cleft via mutation of active-site amino acids improved crystalline PET degradation, indicating that the *Is*PETase was not fully optimised for PET metabolism. This, in conjunction with the initial isolations focusing on amorphous PET (1.9% crystalline) instead of the more crystalline PET abundant in bottle recycling plants (15.7% crystalline; Yoshida et al. [161]) suggests that the origin of the first *I. sakaiensis* isolate from a recycling plant might be coincidental.

Mere changes in polymer mechanical properties and physical structure, even when observed in concert with microbial biomass production, are insufficient evidence to confirm polymer biomineralisation by microbial isolates [172]. Physical losses of plastic mass should also be reported. Plastics can be incorporated into growth media as plastic films, powders or granules, and emulsifications. The first two approaches are primarily used to identify physical changes in polymer structure and the accumulation of biomass as first lines of evidence for plastic degradation (Table 1; Fig. 4). Evidence of polymer degradation from plastic films or polymer granules predominantly requires changes in polymer roughness, the formation of holes or cracks, fragmentation or color changes, confirmed using visual methods such as scanning electron microscopy (SEM), Fourier-transform infrared spectroscopy (FTIR) [173] or atomic force microscopy [174]. However, visual changes in surface structure, changes in plastic mass and mechanical properties do not provide direct evidence of biodegradation [175] because these physical changes cannot be distinguished from abiotic degradation. Where biodegradation is demonstrated it is likely that microbiomes work in conjunction with abiotic factors to impact the structural integrity of polymers [176]. Most polymers are too large to transverse the cell membranes and must be initially depolymerised (e.g. by heat, visible and non-visible spectrum light and oxygen) [177]. Additionally, measuring changes in the surface structure or molecular weight of plastics does not discriminate between the degradation of polymers or their additives [172]. Therefore, in addition to plastic film and granule-infused media, we recommended that biomass accumulation on plastic surfaces and changes to polymer structure should be accompanied by the detection of plastic metabolites to describe growth-linked biodegradation.

A common method for assessing microbial plastic metabolism is by observing clear zones in agar containing emulsified plastic [175, 178]. However, emulsifications are usually limited to amorphous or lower molecular weight plastics while environmental waste plastics such as nylon, PE and PET typically have a higher molecular weight, limiting the analysis of these pollutant plastics. In addition, solvents and surfactants widely used to form plastic emulsions are themselves documented to be degraded by microorganisms [179, 180]. Therefore, observation of clearance zones in culture media containing plastic emulsions should ideally be associated with other empirical tests, such as observations of incorporation of radiolabeled carbon from the polymer backbone into microbial biomass. Because plastic typically comprises the predominant or only carbon source in plastic metabolism assays, only small amounts of evolved CO_2_ are typically required to be detected to indicate polymer metabolism [175]. In addition to CO_2_, other plastic metabolites hypothesised to be produced during plastic degradation (e.g. the production of mono-(2-hydroxyethyl) terephthalate during PET hydrolysis) may be identified using methods such as liquid/light Chromatography-Mass Spectrometry, which detects multiple compounds in a single analytical run [181]. This approach was employed to implicate the role of a putative depolymerase in PHB degradation by *Aspergillus fumigatus* [182]. Similarly, HPLC-mediated detection of the PET-degradation metabolites MHET and terephthalate provided evidence for *Is*PETase involvement in PET degradation [183]. These methods, combined with approaches employed to detect changes in polymer structure and metabolism (Fig. 4) provide powerful evidence for confirming plastic biodegradation.

Knowledge of genes known to be associated with plastic degradation provides a strong tool to identify new degraders and genes among microbiome communities. For instance, Danso, et al. [29] developed a hidden Markov model (HMM) to search genome and metagenome databases for the presence of potential PET hydrolases. The authors used the sequences from nine different enzymes with verified activity on PET-based substrates and identified 504 possible PET hydrolase candidate genes. Studies such as this, and the work of Gambarini, et al. [28], indicates a huge potential for mining molecular databases for plastic degradation-conferring genes (PDGs). One useful approach to verify PDGs experimentally is by heterologous expression of the microbiome-derived candidate genes in a host that lacks degradation capacity in the absence of the introduced gene, followed by confirmation of the plastic-degrading phenotype of the transformant. Heterologous expression in hosts such as *Escherichia coli* has been used to verify plastic degradation-conferring phenotypes of PDGs encoding putative PHB-depolymerases, esterases, cutinases, carboxylesterase and PET hydrolases from a wide variety of bacteria, and some fungi [29, 184–186]. Overexpression in heterologous hosts is also a valuable tool for purifying high levels of enzyme for in vitro assays or studying enzyme crystal structure. Another approach is to disrupt or silence the candidate PDGs in the endogenous background and assess the effect this has on the plastic degradation phenotype. Mining metagenomes using the candidate gene approach does not inform on the discovery of completely novel determinants, or accessory factors that have not been previously described. Under this scenario, genotype-phenotype-based studies of individual degrading strains are still important to identify novel determinants, using methods such as DNA library screens in heterologous hosts, random mutagenesis or differential transcript expression. However, once PDGs are identified, interrogating metagenomes of closely related species for conserved alleles can inform on important residues and functional domains to exploit for genetic enhancement of plastic degradation traits.

## Manipulating microbiomes to enhance rates and extents of plastic degradation

Different strategies may be employed to overcome the challenges of isolating microorganisms capable of efficient and/or fast plastic degradation. For example, higher temperatures can increase the flexibility of both amorphous [187, 188] and crystalline domains of the polymer chain [189–191], thereby improving their accessibility to enzymatic attack [188]. In this regard, thermophile microbiomes represent a promising source of enzymes because they will likely be more thermostable. In one study, the most thermostable enzyme tested (a leaf-branch compost cutinase (LCC) obtained from an uncultured bacterium [186]) had the highest PET depolymerization rates at 65 °C [192]. Degradation rates were further increased after improving enzyme thermostability through site-specific mutagenesis. To date however, only ~ 10% of isolated plastic degradation studies report polymer degradation at temperatures ≥50 °C and only a small fraction (~ 0.5%) of these have been isolated from extreme environments such as hot springs, composts and anaerobic digesters [28]. There would appear to be significant scope for mining thermophile and extremophile microbiomes as a promising source of putative plastic degrading enzymes and microorganisms.

The higher genotypic and phenotypic diversity present in microbial communities compared with single microbial strains may mean that communities are more efficient degraders of xenobiotic pollutants [193]. As such, artificial consortia created by selecting a small number of plastic degrading microorganisms within an already existing consortium (i.e., using a top-down approach [194]), or combining separately isolated microbial strains (i.e., using a bottom-up approach [162]) may be a useful strategy for improving plastic biodegradation. Alternatively, directed mutagenesis to improve gene expression and enzyme function, along with metabolic engineering and synthetic biology tools, could be exploited to obtain more efficient plastic-degrading consortia. Specifically, the introduction or modification of interspecific microbial interactions (such as intercellular communication via metabolite exchange) could be used to create consortia with improved biodegradation traits [195, 196]. Additionally, the segmentation of metabolic pathways among strains such that each organism produces an intermediate compound that can be used by the next organism in the pathway can be used to reduce the metabolic burden on any one organism. Because only limited information is available regarding genes and enzymes involved in plastic biodegradation [28], an improved understanding of degradation pathways by single strains and multi-strain co-degradation pathways is first required to facilitate this approach.

## Conclusions

The impacts of global plastic pollution on microbiomes are diverse, ranging from the direct consequences of toxic leachates on microbial community health and activity to the indirect effects of plastics on host organisms and environments. Many hundreds of microbial species, genes and enzymes are implicated in plastic degradation. For a small number of particularly bio-based plastics, such as PLA, clear evidence is presented for their microbial degradation. However, for the majority of commercial plastics, evidence for microbial degradation remains weak, with studies failing to confirm microbial growth on the synthetic polymer. To ensure the correct identification of plastic-degrading taxa and enzymes, facilitating their improvement by environmental, biotic and genetic manipulation, multiple lines of evidence for plastic degradation should be presented. Ideally this will include evidence of changes in the polymer structure, mass loss and detection of degradation products, along with confirmation of the microbial strain and putative plastic-degrading enzymes and associated genes. Such details are essential for organisms and enzymes capable of plastic degradation to be reliably differentiated from those only capable of degrading the more labile carbon within predominantly amorphous plastics, plastic monomers, fillers and additives.

## Abbreviations

CAM: Contact angle measurement
DRIFT spectroscopy: Diffuse reflectance infrared Fourier transform spectroscopy
DSC: Differential scanning calorimetry
EDS: Energy dispersive spectroscopy
EFM: Epi-fluorescence microscopy
FCM: Flow cytometry
FTIR: Fourier-transform infrared spectroscopy
GC-FID: Gas chromatography with flame ionization detection
GC-MS: Gas chromatography-mass spectrometry
GPC: Gel permeation chromatography
HDPE: High-density polyethylene
HPLC: High-performance liquid chromatography
IR spectroscopy: Infrared spectroscopy
LDPE: Low-density polyethylene
LLDPE: Linear low-density polyethylene
MALDI: Matrix assisted laser desorption/ionization
MHET: Mono-2-hydroxyethyl terephthalate
MHETase: A hydrolase enzyme which cleaves MHET
MS: Mass spectrometry
NMR: Nuclear magnetic resonance
PBAT: Polybutylene adipate terephthalate
PCL: Polycaprolactone
PE: Polyethylene
PEA: Polyesteracetal
PEG: Polyethylene glycol
PES: Polyestersulfone
PESU: Polyethersulfone
PET: Polyethylene terephthalate
PHA: Polyhydroxyalkanoate
PHB: Polyhydroxybutyrate
PHBV: Poly (3-hydroxybutyrate-co-3-hydroxyvalerate)
PHO: Polyhydroxyoctanoate
PLA: Polylactic acid
PMCL: Poly (4-methyl-ε-caprolactone)
PS: Polystyrene
PP: Polypropylene
PU: Polyurethane
PVA: Polyvinyl alcohol
PVC: Polyvinylchloride
QCM-D: Quartz crystal microbalance with dissipation monitoring
RDS: Rheometrics dynamic spectrometer
SEC: Size-exclusion chromatography
SEM: Scanning electron microscopy
TOC: Total organic carbon
TGA: Thermogravimetric analysis
TPA: Terephthalic acid
UPLC: Ultra-performance liquid chromatography
WAXS: Wide angle X-ray scattering
XPS: X-ray photoelectron spectroscopy
XRD: X-ray powder diffraction
